# Physician Perception of Blood Pressure Control and Treatment Behavior in High-Risk Hypertensive Patients: A Cross-Sectional Study

**DOI:** 10.1371/journal.pone.0024569

**Published:** 2011-09-14

**Authors:** José R. Banegas, Krista Lundelin, Mariano de la Figuera, Juan J. de la Cruz, Auxiliadora Graciani, Fernando Rodríguez-Artalejo, Juan García Puig

**Affiliations:** 1 Department of Preventive Medicine and Public Health, Universidad Autónoma de Madrid, Madrid, Spain; 2 Network Biomedical Research Centre in Epidemiology and Public Health (CIBERESP), Ministry of Science and Innovation (Instituto de Salud Carlos III), Madrid, Spain; 3 Instituto de Investigación Hospital Universitario La Paz (IdiPAZ), Madrid, Spain; 4 Vascular Metabolic Unit, Division of Internal Medicine, Hospital Universitario La Paz, Madrid, Spain; 5 Family and Community Medicine Teaching Unit, Sardenya Primary Care Center, Barcelona, Catalonia, Spain; University of British Columbia, Canada

## Abstract

**Objective:**

We examined physician perception of blood pressure control and treatment behavior in patients with previous cardiovascular disease and uncontrolled hypertension as defined by European Guidelines.

**Methods:**

A cross-sectional study was conducted in which 321 primary care physicians throughout Spain consecutively studied 1,614 patients aged ≥18 years who had been diagnosed and treated for hypertension (blood pressure ≥140/90 mmHg), and had suffered a documented cardiovascular event. The mean value of three blood pressure measurements taken using standardized procedures was used for statistical analysis.

**Results:**

Mean blood pressure was 143.4/84.9 mmHg, and only 11.6% of these cardiovascular patients were controlled according to 2007 European Guidelines for Hypertension Management target of <130/80 mmHg. In 702 (49.2%) of the 1426 uncontrolled patients, antihypertensive medication was not changed, and in 480 (68.4%) of these cases this was due to the physicianś judgment that blood pressure was adequately controlled. In 320 (66.7%) of the latter patients, blood pressure was 130–139/80–89 mmHg. Blood pressure level was the main factor associated (inversely) with no change in treatment due to physician perception of adequate control, irrespective of sociodemographic and clinical factors.

**Conclusions:**

Physicians do not change antihypertensive treatment in many uncontrolled cardiovascular patients because they considered it unnecessary, especially when the BP values are only slightly above the guideline target. It is possible that the guidelines may be correct, but there is also the possibility that the care by the physicians is appropriate since BP <130/80 mmHg is hard to achieve, and recent reviews suggest there is insufficient evidence to support such a low BP target.

## Introduction

The patient with cardiovascular disease (CVD) is the most important priority for cardiovascular prevention in clinical practice [1], [2]. Hypertension (HT) is one of the main prognostic factors for CVD [1], [3], but blood pressure (BP) control in hypertensive patients with CVD is suboptimal [4], [5]. Failure of the physician to begin or intensify treatment when the therapeutic goals are not met is a current challenge for research and action according to some authors [6]–[8].

Uncertainty as to whether clinical BP reflects the true value of BP is one of the main reasons for not intensifying therapy in uncontrolled hypertensive patients, an issue that has been systematically examined in diabetics with HT [9]. Other studies have found that one reason physicians say they theoretically would not intensify treatment [10], [11], or actually do not intensify it in their own practice [10], [12], [13], is satisfaction with BP values close to the therapeutic goal. In reality, physicians usually overestimate the degree of BP control in their patients [14]–[16]. Other studies have reported that BP levels at the office visit predict antihypertensive medication change [9], [17]–[19]. But little is known about the factors independently associated with overestimation of BP control, and lack of change in treatment due specifically to that overestimation. So-called competing demands, estimated by length of the office visit or number of problems the patient has, may also be factors that contribute to treatment change for elevated BP [9], or may be alternatives to the conventional reasons for failing to intensify treatment [20].

The high prevalence of treatment inaction in patients with poor BP control [7], [16], [21] suggests that physicians must sometimes have good reasons for not changing treatment, although few studies have addressed this question. It has been reported that “white coat” HT and treatment non-compliance are factors often cited by physicians as contributors to therapeutic inaction in hypothetical patients with elevated BP [22], which could be considered inaction that is clinically appropriate.

For these reasons, the present study examines the clinician perception of BP control and the physician treatment behavior (change or no change in antihypertensive medication) in patients with uncontrolled hypertension. We also examine the main sociodemographic and clinical factors independently associated with these, considering some competing demands as an additional factor. Our study focuses specifically, for the first time, on patients with previous CVD, in whom clinical inaction could have more serious consequences, in the primary care setting, where most hypertensive patients are monitored. The results of this study may provide useful information to improve the quality of care of patients with hypertension.

## Methods

### Ethics statement

The study protocol was approved by the Clinical Research Ethics Committee of the “La Paz” University Hospital in Madrid. All the patients provided written informed consent for the study.

### Research design and methods

The data are taken from a cross-sectional study conducted throughout Spain between April and June 2008. A total of 321 primary care physicians from primary healthcare clinics spread across the geographic regions covered by Spain's national healthcare system participated in the study (participation rate, 94.1%). Physicians were chosen in proportion to the number of inhabitants in each geographic region; and within each region, selection of physicians also took into account their geographic dispersion in outpatient practice lists. Physicianś mean age of 51±7 years, most with over 10 years professional experience and with morning office hours. These physicians consecutively recruited 1,812 patients (maximum of 6 patients per physician) aged 18 years or over, diagnosed with hypertension (BP ≥140/901mmHg) and on anti-hypertensive drug treatment for at least one year, who had suffered at least one of the following cardiovascular events, documented by hospital medical report: ischemic stroke (transient ischemic attack or acute stroke); cerebral hemorrhage; angina or myocardial infarction; coronary revascularization (by-pass, stent); congestive heart failure; or aortofemoral bypass graft surgery [2], [23]. The sample size was predetermined according to the expected frequency of treatment inaction in HT in Spain [16].

A specific case report form was filled out by participating physicians (mostly at the end of the study visit), which included information on their own practice, data to be obtained from the patients, information to be drawn from the medical records, and measurements performed on patients during the study visit. Structured information was collected on the following cardiovascular risk factors: current smoking (daily cigarette consumption at least during the last month), dyslipidemia and diabetes mellitus (type 1 or type 2) recorded in the medical record, obesity (body mass index (BMI) based on measured height and weight ≥30 kg/m^2^), and abdominal obesity (measured waist circumference >102 cm in men and >88 cm in women). Information was also collected from the medical record on target organ damage: left ventricular hypertrophy (Sokolow electrocardiographic criteria: SV1+RV5–6 >38 mm, or Cornell electrocardiographic criteria: SV3+RaVL >28 mm in men and >20 mm in women, or Cornell product >2440 mm/ms, or echocardiogram with left ventricular mass index ≥125 g/m^2^ in men, or ≥110 g/m^2^ in women), arterial wall thickening (in carotid: >0.9 mm or atherosclerotic plaque), slight increase in serum creatinine (men: 1.3–1.5 mg/dl; women: 1.2–1.4 mg/dl), and microalbuminuria (30–300 mg/24h, or albumin/creatinine ratio >22 mg/dl in men and >31 mg/dl in women). Family history of early CVD was also collected [2], [23].

Information on BP and HT was extracted by participating physicians from the medical record: duration, grade of HT at the visit before the study visit (previous HT) [23], and current type of drug treatment (as well treatments related to diet and lifestyle) and their duration. Treatment compliance was estimated by asking the patient: “Most hypertensive patients with complications, like you, take so many medications that they often dońt take some of their pills. Does this happen to you too?”

BP was measured three times during the study visit, at rest, using calibrated mercury sphygmomanometers or validated automatic devices with appropriate cuff size (two sizes), following standardized procedures [23]. The mean of these three measurements was used for the statistical analyses, and controlled HT was defined as BP <130/80 mmHg, which was the therapeutic target in individuals with CVD at the time of the survey [2], [23].

After measuring BP, physicians filled out a questionnaire asking a closed-ended question to report if they considered that their patients' BP was adequately controlled and if they changed the antihypertensive drug treatment at the study visit. If they modified the treatment, they were asked what change had been introduced (increased dosage, added new drug, drug switch, other) and why (lack of drug efficacy, side effects, price, other reasons). Treatment inaction could be defined as no change in treatment despite uncontrolled BP, although this does not necessarily presuppose an inappropriate clinician decision. The information on the lack of change in the treatment was obtained by comparing current treatment as recorded in the medical history and potential change in treatment noted at the visit, and the reasons were ascertained, including the physician's judgment that the patient did not need a change in treatment, postponement of the treatment decision by scheduling the patient for another appointment within 2–3 weeks, referral to the specialist, patient refusal to accept the change, and any other reasons the physician wished to note. Sociodemographic data were also collected on the physicians, as well as number of patients seen per day, length of patient visit, and whether they followed guidelines for management of HT.

### Statistical analysis

Complete information on all the study variables used was available for 1,614 patients. The descriptive results are expressed as absolute frequencies and percentages for the qualitative variables, and as means with their standard deviations for the quantitative ones. The rates of BP control and of treatment change were calculated. We then examined the sociodemographic and clinical characteristics associated with physician perception of adequate BP control, and with treatment change, in uncontrolled hypertensive patients.

Multiple logistic regression models were used to determine whether the following variables were independently associated with the main two reasons of “no treatment change” in uncontrolled hypertensive patients (physician opinion of adequate control, and early scheduling of next appointment): patient age (in years), sex, educational level (no education or primary education; secondary education or university), cardiovascular risk factors such as obesity (yes/no), abdominal obesity (yes/no), smoking (yes/no), dyslipidemia (yes/no), diabetes (yes/no), family history of premature CVD (yes/no), current BP at the office visit (≥160/100; 140–159/90–99; 130–139/80–89 mmHg), previous HT (grade 1 or 140–159/90–99 mmHg; grade 2/3 or ≥160/100 mmHg), duration of HT (years), duration of antihypertensive treatment (years), target organ damage (yes/no), treatment compliance (yes/no), mean number of patients/day, mean consultation time per patient (minutes), and physician compliance with guidelines for HT management. Following the bivariate analyses, criteria of clinical relevance (variables shown to be clinically relevant in the literature) and of statistical significance (bivariate p<0.20) were used to select the variables for the multiple logistic regression. Statistical significance was established at p<0.05 for 2-tailed tests. The analysis was made with SPSS package version 15.0 (SPSS, Chicago, IL, USA).

## Results

Mean patient age was 66.5 (±10.8) years, 62,6% were men, and 27.8% had secondary or higher level education (**Table 1**). Some 39.3% were obese, 64% had abdominal obesity, and 34.4% had diabetes (type 2 in 95% of cases). Half of the patients had target organ damage. Mean duration of HT (from time of diagnosis) was 9.2 years, and mean duration of antihypertensive treatment was 8 years. In the visit before the study visit (95% had visited in the previous three months), 55.3% had grade 1 HT.

**Table 1:** Sociodemographic and clinical characteristics of the study sample.

Variable	
Age, years (SD)	66.5 (±10.8)
Men, %	62.6
Educational level, %	
No education or primary level	72.2
Secondary level or university	27.8
Systolic/diastolic blood pressure at office visit, mmHg (SD)	143.4 (±16.1) / 84.9 (±11.2)
Systolic/diastolic blood pressure <130/80 mmHg, %	11.6
Previous hypertension grade, %	
Grade 1 (140–159/90–99 mmHg)	55.3
Grade 2/3 (≥160/100 mmHg)	44.7
Body mass index, kg/m^2^	29.4 (±4.9)
Waist circumference, cm	101.7 (±15.3)
Obesity, %	39.3
Abdominal obesity, %	64.0
Smoking, %	23.3
Dyslipidemia, %	67.4
Diabetes mellitus, %	34.4
Family history of early CVD, %	43.3
Compliance with drug treatment, %	58.5
Target organ damage, %	53.2
Left ventricular hypertrophy	38.9
Arterial wall thickening	23.0
Mild increase in serum creatinine	25.2
Microalbuminuria	20.4
Associated cardiovascular disease, %	100.0
Ischemic heart disease or coronary revascularization	68.4
Cerebrovascular disease	23.7
Heart failure	17.6
Peripheral artery disease	13.3

CVD indicates cardiovascular disease. See Methods section for definition of risk factors.

All patients were currently being treated with at least one antihypertensive drug: 24.9% in monotherapy, 42.8% with two drugs, and 32.3% with three or more drugs (p<0.001). In all, 58.5% of patients said they were complying with their drug treatment. In addition, a low salt diet had been recommended to 82% of patients, and a low calorie diet to 65.2%.

The participating physicians saw a mean of 46.7±18.6 patients per day, with an average visit time of 7.3±2.9 minutes. About 83% stated that they used guidelines for HT management.

### Blood pressure control and physician perception of control

Mean BP at the study visit (current BP) was 143.4 (±16.1)/84.9 (±11.2) mmHg (**Table 1**). About 69.6% of the 1,426 uncontrolled patients had levels ≥140/90 mmHg, and 30.4% had 130–139/80–89 mmHg (**Table 2**). Control varied from 7.9% in patients with heart failure to 15.6% in those with ischemic heart disease, with better control of diastolic than systolic BP.

**Table 2:** Blood pressure control based on measurement (objective) and according to physician opinion (subjective).

	Objective control		
Subjective control	<130/80 mmHg	≥130/80 mmHg	Total
Yes	185 (11.5%)	570 (35.3%)	755 (46.8%)
No	3 (0.2%)	856 (53.0%)	859 (53.2%)
Total	188 (11.6%)	1426 (88.4%)	1614 (100%)
	**<140/90 mmHg**	≥**140/90 mmHg**	**Total**
Yes	579 (35.9%)	176 (10.9%)	755 (46.8%)
No	43 (2.7%)	816 (50.6%)	859 (53.2%)
Total	622 (38.5%)	992 (61.5%)	1614 (100%)
	**<160/100 mmHg**	**≥160/100 mmHg**	**Total**
Yes	735 (45.5%)	20 (1.2%)	755 (46.8%)
No	559 (34.6%)	300 (18.6%)	859 (53.2%)
Total	1294 (80.2%)	320 (19.8%)	1614 (100%)

BP control according to the physician (subjective control) after evaluating the values measured in the visit was 46.8% (95% CI. 43.2%–50.4%), much higher than the 11.6% of control (95% CI, 7.0%–16.2%) observed when the objective measure was used (**Table 2**). The degree of physician overestimation of objective BP control clearly decreases with increasing threshold for BP control: 35.3% of all hypertensive patients (or 40% of uncontrolled hypertensive patients) if ≥130/80 mmHg, 10.9% if ≥140/90 mmHg, and 1.2% if ≥160/100 mmHg (**Table 2**).

In multivariate analysis, overestimation of control was less likely in patients with higher BP levels above goal (**Table 3**). In addition, abdominal obesity, target organ damage and non-compliance with treatment were associated with less overestimation. The rest of the statistically significant variables in the bivariate analysis (previous HT, smoking and dyslipidemia) lost significance in the multivariate analysis.

**Table 3:** Patient factors associated with physician overestimation of blood pressure control in uncontrolled hypertensive patients, from multivariate logistic analysis.

Patient factor	Odds Ratio (95% CI)	*P*
Blood pressure at the study visit		
140–159/90–99 vs. 130–139/80– 89 mmHg	0.04 (0.02–0.06)	<0.001
≥160/100 vs. 130–139/80–89 mmHg	0.007 (0.003–0.013)	<0.001
Abdominal obesity (yes vs. no)	0.71 (0.51–0.97)	0.035
Target organ damage (yes vs. no)	0.74 (0.55–1.00)	0.050
Treatment compliance (no vs. yes)	0.60 (0.44–0.82)	0.002

### Physician treatment behavior

Physicians maintained the dietary treatment in almost 90% of patients at the visit. The drug regimen was modified in 724 (50.8%) patients with uncontrolled hypertension (**Table 4**), in most cases (90%) by adding a new drug or increasing the dose. Drug treatment was not modified in 702 uncontrolled hypertensive patients (49.2%).

**Table 4:** Physician treatment behavior in uncontrolled hypertensive patients, and its causes.

Therapeutic behavior	N (%)
**Total**	1426
**Change in drug treatment**	724 (50.8%)
Lack of efficacy	615 (85%)
Intolerance/adverse effects	22 (3%)
Price	3 (0.4%)
Other	84 (11.6%)
**No change in drug treatment**	702 (49.2%)
Not necessary (adequate control)	480 (68.4%)
Early appointment scheduled	176 (25.1%)
Referral to specialist	12 (1.7%)
Patient does not accept change	11 (1.6%)
Other	23 (3.2%)

Uncontrolled hypertension: current blood pressure ≥130/80 mmHg.

Adequate control: Physician deems control to be adequate (after examining patient's current blood pressure values).

Early appointment scheduled: Patient scheduled for appointment within 2–3 weeks.

In the 570 uncontrolled hypertensive patients perceived to be controlled (Table 2), the most frequent physician behavior was lack of change in treatment (in 480 patients) due to the belief that the patient did not require a change in treatment (Table 4). This represented 68.4% of all cases of treatment inaction. Treatment was changed in only 90 patients for different reasons. In the 856 hypertensive patients correctly perceived to be uncontrolled (Table 2), the predominant physician behavior was change in treatment (634 patients), in almost all cases (615) because the drug was not considered to be effective (**Table 4**). In the 222 remaining patients correctly classified as uncontrolled, the physicians made no change in treatment, but most of these patients (176) were scheduled for another appointment at an early date (within 2–3 weeks) (**Table 4**).

### Reasons and determinants for lack of change in treatment. Adequate BP control and early next appointment

The frequency of not changing treatment due to physician belief that patients were controlled was moderate (160 cases or 33.3%) in hypertensive patients with elevated current BP at visit (≥140/90 mmHg), and very low (3.9%) if BP was very high (≥160/100 mmHg) (**Table 5**). However, the proportion was considerable (320 cases or 66.7%) when current BP was only mildly elevated (130–139/80–89 mmHg), especially if previous HT was grade 1. In all, these 320 cases account for 45.6% of all cases of treatment inaction. Lack of change in treatment due to scheduling the next appointment within 2–3 weeks was also higher when current BP was marginally or moderately elevated (74.4%) than when it was frankly elevated (≥160/100 mmHg) (25.6%) (**Table 5**).

**Table 5:** Frequency of main reasons for lack of change in antihypertensive treatment, stratified by previous and current blood pressure values.

	Reasons for not changing treatment	
Previous hypertension grade and current blood pressure	Adequate control	Appointment in 2–3 weeks
Previous grade 1 HT and current BP 130–139/80–89 mmHg	229 (47.7%)	30 (17.0%)
Previous grade 2/3 HT and current BP 130–139/80–89 mmHg	91 (19.0%)	19 (10.8%)
Previous grade 1 HT and current BP 140–159/90–99 mmHg	69 (14.4%)	46 (26.1%)
Previous grade 2/3 HT and current BP 140–159/90–99 mmHg	72 (15.0%)	36 (20.5%)
Previous grade 1 HT and current BP ≥160/100 mmHg	3 (0.6%)	9 (5.1%)
Previous grade 2/3 HT 3 and current BP ≥160/100 mmHg	16 (3.3%)	36 (20.5%)
Total	480 (100%)	176 (100%)

Not changing treatment: Physician does not change drug treatment in patient with uncontrolled hypertension.

Previous HT: Hypertension grade at visit before the study visit.

Current BP: Blood pressure at the study visit.

**Table 6** shows only those variables that remained statistically significant in the multivariate analysis. Current BP was the factor with the strongest independent association with lack of treatment change due to physician perception of adequate BP control. Compared to patients with BP 130–139/80–89 mmHg at the visit, treatment inaction was less likely in those with BP 140–159/90–99 mmHg (OR, 0.11; 95% CI 0.08 to 0.15), and much less likely in patients with more elevated BP (≥160/100 mmHg) (Table 6). The frequency of treatment inaction was also lower in patients with abdominal obesity, with target organ damage, or in non-compliant patients, irrespective of current BP levels. Previous grade of HT and other variables significantly associated with treatment inaction in the bivariate analysis, lost significance in the multivariate analysis. Finally, in the multivariate analysis, the frequency of not changing treatment due to early next appointment (in 2–3 weeks) was highest in patients with abdominal obesity or target organ damage.

**Table 6:** Factors associated with lack of change in treatment in uncontrolled hypertensives patients, by the main reasons asserted by physicians, from multivariate logistic analysis.

Reason for not changing treatment	Odds Ratio (95% CI)	*P*
**Adequate control**		
Blood pressure at the study visit		
140–159/90–99 vs. 130–139/80–89 mmHg	0.11 (0.08–0.15)	<0.001
≥160/100 vs. 130–139/80–89 mmHg	0.02 (0.01–0.04)	<0.001
Abdominal obesity (yes vs. no)	0.63 (0.47–0.85)	0.003
Target organ damage (yes vs. no)	0.67 (0.50–0.90)	0.007
Treatment compliance (no vs. yes)	0.65 (0.48–0.87)	0.004
**Early appointment scheduled**		
Blood pressure at the study visit		
140–159/90–99 vs. 130–139/80–89 mmHg	1.11 (1.03–1.13)	0.012
≥160/100 vs. 130–139/80–89 mmHg	1.13 (1.12–1.14)	0.015
Abdominal obesity (yes vs. no)	1.25 (1.01–1.50)	0.048
Target organ damage (yes vs. no)	1.46 (1.10–1.93)	0.008

Adequate control indicates that patient does not require change in antihypertensive medication because physician deems control to be adequate (after examining patient's current blood pressure values);

Early appointment scheduled: Physician does not change treatment because patient scheduled for appointment within 2–3 weeks.

## Discussion

Physicians overestimate the level of BP control in hypertensive patients: 40% of patients who in reality have uncontrolled hypertension are perceived as being adequately controlled. The difference between objective BP control and control as perceived by physicians has also been observed in other studies [14]–[16], [24]. Furthermore, the magnitude of treatment inaction in our study was almost 50%, similar to that reported in other studies in primary care [24], and lower than that observed in some other studies [7], [16], including those found in cardiovascular patients in specialty care [21]. However, the prevalence of inaction varies depending on the different methodologies and populations in the various studies [7], [9]–[13], [16]–[18], [21], [24], [25]. Interestingly, factors predictive of overestimation of control and of lack of change in treatment due to misperception of BP control are consistent. In patients who have more elevated BP levels, are obese, or do not comply with treatment – that is, those with higher cardiovascular risk – physicians tend to be less permissive; in these cases physicians are less likely to overestimate the level of control and are more likely to treat more intensely.

### Reasons for not changing treatment

The lack of change in treatment by physicians in uncontrolled hypertensive patients does not appear to be primarily a problem of knowledge of clinical practice guidelines, since most physicians stated that they knew them. In fact, physicians were informed in the study protocol about the appropriate BP target in high-risk patients at work at the time of the survey (<130/80 mmHg; 2007 European guidelines) [2], [23]. However, physicians did not implement the guidelines with respect to this “demanding” treatment targets in cardiovascular patients. BP control achieved under this target was very low, physicians made changes in treatment in only a half of uncontrolled hypertensive patients, and lack of treatment changes occurred in those at only moderately elevated BP values.

Several reasons may explain these findings. First, physicians may be uncertain about the levels of clinical BP, especially when they are only slightly elevated [9], [19]. In our study, a substantial proportion of cases of absence of treatment change in uncontrolled hypertensive patients occurred when BP values were 130–139/80–89 mmHg, near the therapeutic target in patients with previous CVD (<130/80 mmHg). Also, the physicians who chose to have the patient return in 2 to 3 weeks were possibly taking the most appropriate action as they were most likely aware of the inaccuracy and variability of BP measurements. In fact, physicianś lack of treatment change due to appointment in 2–3 weeks was more frequent when patients had slightly elevated current BP than when they had frankly elevated current BP levels.

Second, it must be considered that a guideline-based low BP goal in CVD patients (say <130/80 mmHg) is not consistently supported by trial evidence, as shown after performance of this survey [26], [27]. Thus, many physicians may be sceptical of guidelines as they are frequently based primarily on expert opinion and subject to bias and conflicts of interest. Likewise, our results also suggest that many physicians consider that small elevations of visit BP levels above goal are not of concern and pose little risk to patient health. In fact, the trial-based differences in achieved cardiovascular protection within this range of BP values seem to be small at best [27]. BP levels much higher than the target, however, are associated with increased clinical action.

Third, BP control under 130/80 mmHg is possibly difficult to achieve in many patients. In major trials it has been shown that a target of <140/90 mmHg is only achieved in approximately 60% of patients despite use of 3 or more drugs [28]. In fact, we obtained a quite low (11.6%) rate of BP control in CVD patients under the stringent target <130/80. This makes the concept of putting blame on the physicians inappropriate.

Fourth, of 702 uncontrolled patients in whom physicians did not change treatment, 216 (30.8%) were receiving three or more antihypertensive drugs. In most of these cases, patients were considered by physicians at adequate BP control or had an early next appointment. Although we did not directly ask for number of drugs prescribed as a potential reason for not changing therapy, it could be suggested that it was an additional reason. Fifth, inaction is not explained by physicians having taken additional non-drug measures because in most patients they continue the same dietary and lifestyle recommendations as before the visit. However, treatment compliance does affect inaction, since physicians were more likely to change treatment in non-compliers. Finally, the influence on inaction of competing demands, or problems that most worry the physician in the visit, has varied in different studies [9], [20]. In our study, neither the length of the visit nor patient comorbidities (considered indirectly through the number of drugs taken for other conditions – data not shown), were significantly associated with inaction in the multivariate analysis.

We are not prejudging what the correct therapeutic decisions are in any particular case. However, caution is needed if BP levels are clearly elevated (≥140/90 mmHg, and especially if ≥160/90 mmHg), which are less consistent with clinical uncertainty and in which the associated cardiovascular risk is appreciable.

### Methodological aspects

The study patients are reasonably representative of the hypertensive population attended in primary care centers in Spain [29]. Moreover, the high response rate (89.1%) minimizes selection bias, and we used specific and detailed information from physicians, patients, and medical records. There is a predominance of males, but this reflects what is found in actual clinical practice with patients with cardiovascular disease. The extent to which our findings are generalizable to other populations is uncertain, although overall rates of BP control (<140/90 mmHg) in our study population were similar to those obtained in studies in other countries [4], [5]. Moreover, these limitations would not necessarily affect the associations found between treatment change and its predictive factors.

Furthermore, we decided to categorize patient BP levels, given the pragmatic clinical definition of HT and its control, that is, given usual medical behavior in clinical practice.

Patient compliance with therapy, which could explain some cases of lack of change in treatment, was not considered in detail in our study. However, some studies have shown that questionnaires can reasonably identify non-compliant patients [30].

Although obtaining information on therapeutic behavior during the study visit could increase treatment change, this would result in an underestimate of the proportion of treatment inaction observed. Also, although the type of visit in our study was not specifically for HT monitoring, and this may have somewhat overestimated treatment inaction, the reason for most patient visits was monitoring of their HT.

Finally, neither ambulatory nor self-measured data on BP were available, thus treatment inaction might be partially explained by physicianś belief that actual BP was lower than BP measured at the office (white-coat effect).

### Conclusion

Physicians generally do not comply with BP guidelines concerning application of very low BP target in CVD patients. Physicians do not change antihypertensive treatment in many uncontrolled hypertensive patients because they considered it unnecessary or because they scheduled an early appointment, especially when the BP values are only moderately elevated.

Overall, it is possible that the guidelines may be correct, but there is also the possibility that the care by the physicians is entirely correct or that the patients who have BPs <130/80 mmHg are being overtreated and being put unnecessarily at risk. In fact, the risk for all-cause death and myocardial infarction progressively increases with low diastolic BP, and excessive reduction in diastolic pressure should be avoided in patients with coronary artery disease who are being treated for hypertension [31].

New studies are needed on the social and health consequences of greater BP permissiveness and to clarify the most scientifically appropriate therapeutic targets that are feasible in medical practice [26], [27]. Our finding of inaction that is probably appropriate in those with mildly elevated BP levels would be an additional argument in favor of recent proposals of less stringent therapeutic targets in high-risk hypertensive patients [26], [27]. Finally, additional prospective data (our study was cross-sectional) are also needed on factors that act as barriers to intensification of therapy over time.
